# Performance Assessment of Internal Quality Control (IQC) Products in Blood Transfusion Compatibility Testing in China

**DOI:** 10.1371/journal.pone.0141145

**Published:** 2015-10-21

**Authors:** Gui-Ping Xu, Li-Fang Wu, Jing-Jing Li, Qi Gao, Zhi-Dong Liu, Qiong-Hua Kang, Yi-Jun Hou, Luo-Chuan Zhang, Xiao-Mei Hu, Jie Li, Juan Zhang

**Affiliations:** 1 Transfusion Department, the Second Hospital Affiliated to Chongqing Medical University, Chongqing, China; 2 The Department of Laboratory Medicine, the Second Hospital Affiliated to Chongqing Medical University, Chongqing, China; Providence VA Medical Center and Brown University, UNITED STATES

## Abstract

Internal quality control (IQC) is a critical component of laboratory quality management, and IQC products can determine the reliability of testing results. In China, given the fact that most blood transfusion compatibility laboratories do not employ IQC products or do so minimally, there is a lack of uniform and standardized IQC methods. To explore the reliability of IQC products and methods, we studied 697 results from IQC samples in our laboratory from 2012 to 2014. The results showed that the sensitivity and specificity of the IQCs in anti-B testing were 100% and 99.7%, respectively. The sensitivity and specificity of the IQCs in forward blood typing, anti-A testing, irregular antibody screening, and cross-matching were all 100%. The reliability analysis indicated that 97% of anti-B testing results were at a 99% confidence level, and 99.9% of forward blood typing, anti-A testing, irregular antibody screening, and cross-matching results were at a 99% confidence level. Therefore, our IQC products and methods are highly sensitive, specific, and reliable. Our study paves the way for the establishment of a uniform and standardized IQC method for pre-transfusion compatibility testing in China and other parts of the world.

## Introduction

A blood transfusion is the process of receiving blood or blood components into one’s venous circulation. Though the transfusion of human blood was successfully performed by British obstetrician Dr. James Blundell in the early nineteenth century, this was regarded as a risky and dubious therapy, and many early transfusions resulted in patient deaths [1]. In 1901, the discovery of the ABO blood group system by Karl Landsteiner provided a scientific basis for blood transfusions [2]. Many other blood groups, such as MNS and Rh, have since been discovered [3, 4]. Because of the decrease in transfusion-related infectious diseases and the development of pre-transfusion compatibility testing, such as blood typing, irregular antibody screening, and cross-matching in blood transfusion laboratories, blood transfusions are now much safer than they were in the past [5, 6]. However, transfusion-related deaths still occasionally occur [5]. Blood type-incompatible transfusion is one of the leading causes of transfusion-related deaths. The incidence of ABO-incompatible transfusion is estimated to be 1:38,000 to 1:100,000 units of RBCs in the United States, 1:16,500 to 1:100,000 units of RBCs in the United Kingdom, and around 1:100,000 units of RBCs in Canada [7–10]. To date, because of the lack of hemovigilance systems to monitor and record adverse transfusion events, no statistical data has been seen in China. It was reported that blood type-incompatible transfusions were generally caused by administration errors, around 30% of which occur in blood transfusion laboratories [11, 12]. SHOT’s 2014 Annual Report indicated that 12 ABO-incompatible transfusions were reported that year in the UK, in seven cases, the errors occurred in blood transfusion laboratories [13]. Hence, it is necessary to reinforce quality management in laboratories and to develop internal quality control (IQC) for pre-transfusion compatibility testing in order to avoid these errors [14, 15]. At present, IQC is required in the laboratories of several countries, including China, the UK, and the US [16–18].

However, pre-transfusion compatibility testing is a qualitative or semi-quantitative assay, and its results are mainly obtained by evaluating the intensity of RBC agglutination or comparing the results with standard images using the naked eye. Furthermore, the results of pre-transfusion compatibility testing do not show a Gaussian distribution, which means that the cut-off value used in traditional immunological tests is incapable of determining negative or positive results [19]. Therefore, special IQC products and methods need to be developed. Though some commercial IQC products are available, such as WBcorQC from Immucor, AlbaQ-Chek from Ortho, DG Gel Control from Diana, and the Pelicheck panel from Sanquin, these products do not apply to cross-matching and therefore they cannot be relied upon to ensure comprehensive IQC. At present, most studies focus on external quality assessment in transfusion laboratories; studies on the IQC of pre-transfusion compatibility testing in laboratories remains rare [20–22]. Uniform and standardized IQC products and methods have not yet been developed for pre-transfusion compatibility testing in China [19, 23]. In this paper, we present the IQC tests that were conducted in our laboratory from 2012 to 2014, and we analyze the sensitivity, specificity, and confidence levels of these products. By evaluating the reliability of our IQC method, our study can contribute to establishing a standardized IQC method for pre-transfusion compatibility testing in China and around the world.

## Materials and Methods

### 1. Cells and reagents

Blood typing, cross-matching IQC products, and ABO cells were purchased from Kinghawk Pharmaceutical Company (Beijing, China). Antibody screening IQC products and screening panel cells were purchased from Li Bo Pharmaceutical Biotechnology Company (Jiangyin, China). Anti-A, anti-B, and anti-D IgM monoclonal antibodies were purchased from Hemo-Pharmaceutical & Biological Company (Shanghai, China). All experiments were carried out by the micro-column gel method using the WADiana Automatic Blood Group Analyzer (GRIFOLS, Spain) in the Transfusion Department of the Second Hospital Affiliated to Chongqing Medical University during the 2012–2014 period. The IQC products used were composite blood samples tested once daily prior to routine pre-transfusion compatibility testing. Each of the 697 IQC samples for blood typing, irregular antibody screening, and cross-matching were tested and analyzed.

### 2. ABO and RhD typing

The IQC products for blood typing were samples IQC1 and IQC2, mixed by the manufacturer, which together constitute a complete QC system for blood typing and antibody screening. IQC1 contained RBCs of type A and was RhD-positive, while IQC2 contained RBCs of type B and was RhD-negative. In forward ABO and RhD typing, a 10 μL 5% RBC suspension, which was diluted with sterile normal saline (NS), was added to the A, B, D, and Ctrl reaction columns of the DG Gel ABO-CDE card (GRIFOLS, Spain). For reverse ABO blood typing, 50 μL of 0.8% type A and B RBCs were added to the N/A1 and N/B reaction columns of the DG Gel ABO-CDE card, respectively, and 50μL of IQC1 and IQC2 plasma were then added to each column. Finally, the cards were centrifuged at 1,500 rpm for nine minutes to indicate the results.

### 3. Antibody screening

Antibody screening and blood typing used the same IQC products, IQC1 and IQC2, both of which contained the anti-D antibody. IQC1 served as the weak antibody control, and 50 μL of 1% RBC samples were added to DG Gel Coombs (GRIFOLS, Spain), followed by the addition of 25 μL of IQC plasma to each column. The cards were then incubated at 37°C for 15 minutes and centrifuged at 1,500 rpm for nine minutes to indicate the results.

### 4. Cross-matching

IQC products for cross-matching were made up of six IQC blood batches from No. 1 to No. 6, which were different than those used for blood typing and antibody screening. No. 1 with blood type A and RhD-negative and No. 4 with blood type A and RhD-positive served as recipient samples, which were respectively used to detect IgG and IgM antibodies. No. 2 with blood type A and RhD-negative and No. 3 with blood type A and RhD-positive served as donor samples for No.1, which contained the IgG anti-D antibody with 1:16 titer. No. 5 with blood type B and RhD-positive and No. 6 with blood type O and Rh(D) served as donor samples for No. 4. The major column contained 50 μL of the donor’s 1% RBCs and 25 μL of the recipient’s plasma. The minor column contained 50 μL of the recipient’s 1% RBCs and 25 μL of the donor’s plasma. The cards were incubated at 37°C for 15 minutes and then centrifuged at 1500 rpm for nine minutes to show the results.

### 5. Evaluation of quality control and statistical analysis

When the difference between the value of the agglutination intensity of the IQC testing results and the reference value was no higher than 1, and no false-positive results were found, the test was defined as “under control”. Otherwise, it was defined as “out of control” [19]. Sensitivity, specificity, and reliability were used to evaluate the results of the IQC products [16, 24]. Sensitivity was defined as the percentage of true-positive results in all positive tests. Specificity was defined as the percentage of true-negative results in all negative tests. Confidence level was analyzed using binomial distribution as suggested by the American Association of Blood Banks (AABB) technical manual [16]; the confidence level was calculated using function BINOMDIST (No. passed-1, total IQC numbers, 0.95 or 0.99, TRUE) in MS Office’s Excel software.

## Results

### 1. IQC for blood typing

Blood type is a heritable antigen expressed on RBC surfaces; these antigens may be glycoproteins (such as the ABH, Lewis, Ii, and P blood group systems) or polypeptides (such as the Rh, MNS, Kidd, and Kell blood group systems), which usually show individual differences [16]. Blood typing is a test that detects the antigens on RBC surfaces and the antibodies in serum through visualized RBC agglutination reactions. ABO and RhD are the most important blood types [25, 26]. In all 697 results, the IQCs in the ABO, RhD antigen, and anti-A antibody testing were fully under control. Sensitivity and specificity were both 100%. The IQC results in anti-B antibody testing were out of control twice, when false-positive results occurred. Sensitivity and specificity were 100% and 99.7%, respectively. The reliability analysis showed that 100% and 97% of IQC results in anti-B testing were at confidence levels of 95% and 99%, respectively. For the ABO, RhD antigen, and anti-A antibody testing, 100% and 99.9% of the results were at confidence levels of 95% and 99%, respectively (Table 1). Overall, the IQC blood typing results showed a 95% confidence level in our laboratory.

**Table 1:** Analysis of IQC for blood typing.

Blood type		Reference value^c^	Test No. ^c^	QC passed^c^	QC failed^c^	Sensitivity (%)	Specificity(%)	95% Confidence level	99% Confidence level
ABO	A Ag^a^	4+/-	697/697	697/697	0/0	100	100	100%	99.9%
	B Ag^a^	-/4+	697/697	697/697	0/0	100	100	100%	99.9%
	Anit-A^b^	-/4+	697/697	697/697	0/0	100	100	100%	99.9%
	Anti-B^b^	4+/-	697/697	697/695	0/2	100	99.7	100%	97.0%
Rh	D Ag^a^	4+/-	697/697	697/697	0/0	100	100	100%	99.9%

^a^ Blood group antigens for forward blood typing.

^b^Blood group antibodies for reverse blood typing.

^c^QC1/QC2.

### 2. IQC for irregular antibody screening

Irregular antibody screening is used to detect unexpected antibodies in the serum of patients; this screening could detect clinically significant antibodies with the exception of regular antibodies anti-A and anti-B. The patient’s serum is tested against screening cells; once an irregular antibody is detected, its specificity can be further identified using a panel of type O reagent RBCs of known antigen composition. After the identification of the specific antibody, antigen-negative blood can be prepared for cross-matching and transfusion. Irregular antibody screening is a routine pre-transfusion test that can detect 99.9% of irregular antibodies in patients’ serum [17, 25]. When analyzing our 697 samples, we found that all of the IQC results for antibody screening were under control. Sensitivity and specificity were both 100%, and 100% and 99.9% of the results were at confidence levels of 95% and 99%, respectively (Table 2).

**Table 2:** Analysis of IQC for irregular antibody screening.

Panel cells	Reference value^a^	Test No.^a^	QC passed^a^	QC failed^a^	Sensitivity(%)	Specificity(%)	95% Confidence level	99% Confidence level
cells 1	2+/±	697/697	697/697	0/0	100	100	100%	99.9%
cells 2	2+/±	697/697	697/697	0/0	100	100	100%	99.9%
cells 3	-/-	697/697	697/697	0/0	100	100	100%	99.9%

^a^ QC1/QC2.

### 3. IQC for cross-matching

Cross-matching is a serological test for evaluating compatibility between blood donors and recipients. IQC procedures are able to ensure the sensitivity of the tests to avoid masked or undetected antibodies in antibody screening from being missed [17]. All 697 results for IQC in cross-matching testing were under control. Sensitivity and specificity for cross-matching were both 100%, and 100% and 99.9% cross-matching results had 95% and 99% confidence levels, respectively (Table 3).

**Table 3:** Analysis of IQC for cross-matching.

Donor	Recipient	Reference value^a^	Test No.^a^	QC passed^a^	QC failed^a^	Sensitivity (%)	Specificity (%)	95% Confidence level	99% Confidence level
1	2	-/-	697/697	697/697	0/0	100	100	100%	99.9%
1	3	2+/-	697/697	697/697	0/0	100	100	100%	99.9%
4	5	4+/4+	697/697	697/697	0/0	100	100	100%	99.9%
4	6	-/4+	697/697	697/697	0/0	100	100	100%	99.9%

^a^ major/minor.

## Discussion

In the past, infectious disease was the main risk of receiving blood components [27]. Along with technological innovations, the incidence of infectious diseases caused by blood transfusions has decreased significantly [6, 28]. Although blood transfusions are becoming safer than ever due to the lowered incidence of infection, this procedure still carries many potential risks. The most serious risk is an acute hemolytic transfusion reaction caused by blood type-incompatible transfusion [27, 28]. According to a recent report by the United States Food and Drug Administration (FDA), ABO-incompatible transfusions account for 10% of all transfusion-related deaths [6]. Most blood type-incompatible transfusions result from human errors [13], with 10% of these arising through incorrect medical prescriptions, 40% in blood banks, and 50% in pre-transfusion compatibility testing [29]. Errors outside of laboratories could be dramatically decreased by applying new technologies, such as barcodes and radiofrequency identification [27]. According to SHOT’s 2014 Annual Report, manual methods are more likely to lead to laboratory errors than automated ones [13, 30]. In order to reduce mistakes in blood transfusion laboratories, sensitive, standardized, and automated testing approaches, such as the micro-column gel method, are highly recommended [31]. It was reported by the UK National External Quality Assessment Scheme (UK NEQAS) that the micro-column gel method is used for 65% of ABO typing and 90% of antibody screening [32]. The results of the College of American Pathologists (CAP) Transfusion Medicine J-survey showed that gel methods had gradually increased yearly between 2005 and 2010 in the US [26].

Employing IQCs is another important strategy for avoiding and reducing laboratory errors. IQCs can assess the entire testing process, including the use of reagents, instruments, and all other factors involved, ensuring the reliability of test results. Hence, IQC products are indispensable in transfusion compatibility laboratories, as demanded by many organizations, such as the AABB, the British Committee for Standards in Hematology (BCSH), and the Ministry of Health (MOH) of the People's Republic of China [16–18]. However, due to the lack of uniform methods and standards for IQCs and cost-saving concerns, most transfusion compatibility laboratories in China have not yet developed methods for IQC [19].

By analyzing the 697 results in our laboratory, we found that the sensitivity and specificity of the IQCs in anti-B testing were 100% and 99.7%, respectively, and those for forward blood typing, anti-A testing, irregular antibody screening, and cross-matching were all 100%. Reliability analysis showed that 97% of anti-B results were at a 99% confidence level, while 99.9% of forward blood typing, anti-A testing, irregular antibody screening, and cross-matching results were at a 99% confidence level. This suggests that our IQC method was able to ensure 97% of IQC results at a confidence level of at least 99%. In this study, false-positive results occurred twice in the IQCs for anti-B reverse typing. The results did not come back under control until the RBC product for reverse typing was replaced by a new one. This suggested that the two false-positive results could have been caused by approaching the RBCs’ expiration date. The shelf-life of RBC products for reverse typing was short. When we received the IQC products, the expiration date was usually less than one month away. Furthermore, because the micro-column gel cards had a relatively closed and stable environment compared with RBC reagents, the IQC products were more prone to external influence. The most common impacts were hemolysis resulting from improper transport and preservation conditions, and repeated restoration to room temperature as a result of frequent testing. Another common variation was contamination resulting from unsterile water or pipette tips, or from incomplete washing in the machine. Hemolysis and contamination could lead to false-positive results. In addition, insufficient restoration to room temperature could weaken agglutination intensity. Air bubbles in the gel could also interfere with the interpretation by the automated machine. It is recommended to centrifuge the gel cards before testing and to reassess unlikely results with the naked eye.

IQCs play an important role in transfusion safety. In our study, 97% of IQC results in blood compatibility testing were at a 99% confidence level, and no error was found in the blood compatibility tests, suggesting that the IQCs could guarantee testing reliability. In our opinion, the ideal IQC products for pre-transfusion testing should contain all irregular antibodies. However, some antibodies occur infrequently. In three years, we only found eight kinds of antibodies (data from our unpublished paper in *Laboratory Medicine and Clinic*), of which the top five antibodies were anti-D, anti-E, anti-M, anti-c, and anti-Le^a^. Given that rare antibodies are usually more expensive to acquire, we recommend adopting a compromise for IQC products; for instance, they might only contain the most prevalent antibodies. Furthermore, these should differ according to their prevalence in certain countries and/or districts. For example, in China, these IQC products might have our top five antibodies, which represent more than 90% of irregular antibodies. In fact, the IQC products for antibody screening from Diamed, Ortho, and Diana only have two kinds of irregular antibodies.

The IQC products we use in this study are still imperfect. Firstly, the irregular antibodies in our IQC products remain simplex, which means we cannot ensure that some irregular antibodies are not missed. Secondly, the IQC products for cross-matching lack weak antibody control. Although a self-diluted weak positive sample is used ever in our laboratory, the result is not stable, and a self-made sample is difficult to trace and unify. Manufacturers are therefore encouraged and advised to develop a suitable product [33]. Our present work is only a preliminary investigation; improved IQC products and IQC methods need to be studied further. Nonetheless, our research paves the way for the establishment of a uniform and standardized IQC method for transfusion compatibility testing in China and other parts of the world.
